# Engineered *Lactobacillus paracasei* Producing Palmitoylethanolamide (PEA) Prevents Colitis in Mice

**DOI:** 10.3390/ijms22062945

**Published:** 2021-03-14

**Authors:** Giuseppe Esposito, Marcella Pesce, Luisa Seguella, Jie Lu, Chiara Corpetti, Alessandro Del Re, Fatima Domenica Elisa De Palma, Giovanni Esposito, Walter Sanseverino, Giovanni Sarnelli

**Affiliations:** 1 Department of Physiology and Pharmacology “V. Erspamer”, Sapienza University of Rome, 00185 Rome, Italy; giuseppe.esposito@uniroma1.it (G.E.); luisa.seguella@uniroma.it (L.S.); chiara.corpetti@uniroma1.it (C.C.); alessandro.delre.phd@gmail.com (A.D.R.); 2 Nextbiomics S.R.L., 80100 Naples, Italy; wsanseverino@sequentiabiotech.com; 3 Department of Clinical Medicine and Surgery, University of Naples “Federico II”, 80131 Naples, Italy; mapesc@hotmail.com; 4 Department of Anatomy and Cell Biology, China Medical University, Shenyang City, Liaoning 110122, China; lvjie@cmu.edu.cn; 5 CEINGE-Biotecnologie Avanzate s.c.a r.l., Department of Molecular Medicine and Medical Biotechnologies, University of Naples Federico II, 80131 Naples, Italy; depalma@ceinge.unina.it; 6 Department of Advanced Biomedical Sciences, University of Naples Federico II, 80131 Naples, Italy; espgiov@unina.it; 7 UNESCO Chair Staff Member, University of Naples “Federico II”, 80100 Naples, Italy

**Keywords:** inflammatory bowel disease, palmitoylethanolamide, ulcerative colitis, probiotic, *Lactobacillus paracasei*, pharmacotherapy

## Abstract

Palmitoylethanolamide (PEA) is an *N*-acylethanolamide produced on-demand by the enzyme *N*-acylphosphatidylethanolamine-preferring phospholipase D (NAPE-PLD). Being a key member of the larger family of bioactive autacoid local injury antagonist amides (ALIAmides), PEA significantly improves the clinical and histopathological stigmata in models of ulcerative colitis (UC). Despite its safety profile, high PEA doses are required in vivo to exert its therapeutic activity; therefore, PEA has been tested only in animals or human biopsy samples, to date. To overcome these limitations, we developed an NAPE-PLD-expressing *Lactobacillus paracasei F19* (pNAPE-LP), able to produce PEA under the boost of ultra-low palmitate supply, and investigated its therapeutic potential in a murine model of UC. The coadministration of pNAPE-LP and palmitate led to a time-dependent release of PEA, resulting in a significant amelioration of the clinical and histological damage score, with a significantly reduced neutrophil infiltration, lower expression and release of pro-inflammatory cytokines and oxidative stress markers, and a markedly improved epithelial barrier integrity. We concluded that pNAPE-LP with ultra-low palmitate supply stands as a new method to increase the in situ intestinal delivery of PEA and as a new therapeutic able of controlling intestinal inflammation in inflammatory bowel disease.

## 1. Introduction

Palmitoylethanolamide (PEA) is a naturally-produced lipid derived from the hydrolysis of its phospholipid precursor, by *N*-acylphosphatidylethanolamine-specific phospholipase D (NAPE-PLD) [1,2,3]. PEA belongs to the larger family of bioactive autacoid local injury antagonist amides (ALIAmides), whose production is induced on-demand by several cells’ types and tissues, during inflammatory noxae [4]. 

PEA exerts potent anti-inflammatory effects, and it has been shown to improve intestinal inflammation, following both intraperitoneal and oral administration [5], in animal models of colitis. More importantly, its efficacy has also been demonstrated in mucosal biopsies from patients with ulcerative colitis (UC) [6,7,8], with the peroxisome proliferator activated receptor α (PPARα) being one of the key receptors mediating these effects [9]. 

Inflammatory bowel disease (IBD), which comprises Crohn’s disease and UC, is a chronic relapsing inflammatory bowel disorder with multifactorial pathophysiology, featuring diarrhea, abdominal pain, and weight loss [10]. In IBD, an altered PEA turnover with relative down-expression of NAPE-PLD and overexpression of its degrading enzymes led to the postulation of an impairment of the acylethanolamide–PPARα anti-inflammatory axis in patients with active UC [11].

In spite of the widespread use of PEA-based over-the-counter preparations for disorders featuring pain and hyper-inflammation [12], and the lack of recorded serious adverse drug reactions [13], its use in treating intestinal inflammatory conditions is currently limited by the high doses required to achieve its therapeutic effect, following oral administration. This strongly limits PEA use in current clinical practice, and alternative strategies to efficiently increase PEA bioavailability are currently under development. 

An innovative approach that may overcome such limitations could be the topical delivery of PEA at the colonic mucosa surface, by genetically-modified probiotics, able to achieve a controlled production of anti-inflammatory molecules. This probiotic system could adhere to the intestinal surface and produce specific bioactive metabolite(s) in response to an exogenous substrate; thus, behaving as a resident “cell factory” for intestinal therapeutics against IBD. This approach was first explored in the pioneer works by Djordjevic and Klaenhammer and Steidler et al. [14,15] in the late 1990s and early 2000s, and was proven to be feasible both in animals and in phase I clinical studies involving IBD patients. 

Using *Lactobacillus paracasei* subsp. *paracasei F19* (pLP) engineered with human *N*-acylphosphatidylethanolamine-specific phospholipase D-(NAPE-PLD) gene, we aimed at generating an in situ drug-delivery probiotic system, able to selectively release PEA in the gastrointestinal (GI) tract, under the boost of ultra-low doses of exogenous palmitate. Previous in vivo studies demonstrated that *Lactobacillus* F19 survived well through the human GI tract and was detected in reasonable numbers in stool specimens from 100% of studied subjects [16]. 

Given the high genetic stability of this widely used probiotic, we tested whether the transformed NAPE-expressing LP (pNAPE-LP) was able to release PEA effectively both in vitro and in vivo, and assessed the in vivo effects of orally administered pNAPE-LP on (i) colitis severity, (ii) plasmatic release of pro-inflammatory signaling molecules and cytokines (iii) mucosal inflammation and neutrophil infiltration and (iv) epithelial barrier integrity in a well-validate murine model of acute colitis. Dextran sodium sulphate (DSS) is a widely used method to study various clinical and histopathological features that reflect those observed in human ulcerative colitis, because of its simplicity, inexpensiveness, and reproducibility [17]. 

## 2. Results

### 2.1. Time-Dependent Production of PEA by pNAPE-LP and Exogenous Palmitate

**Figure 1:**
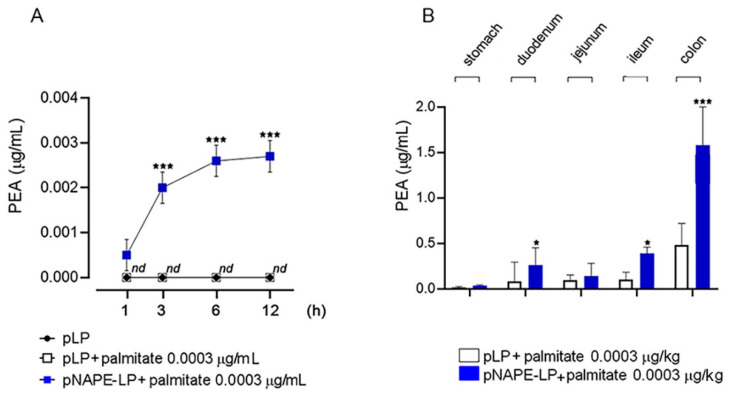
Palmitoylethanolamide (PEA) is time-dependently released by engineered NAPE-LP probiotic under palmitate boost. (**A**) Released PEA levels were evaluated in bacterial supernatant at 1, 3, 6, and 12 h by HPLC–MS and the results are expressed as the mean ± SD of *n* = 4 experiments performed in triplicate. In comparison with pLP in absence of palmitate supply, exogenous palmitate (0.0003 μg/mL) time-dependently increased PEA release from pNAPE-LP probiotics, both *** *p* < 0.001 vs. pLP and pLP in presence of palmitate 0.0003 μg/mL. No detectable amount of PEA was revealed by pLP even in the presence of 0.0003 μg/mL supplementation of exogenous palmitate. (**B**) PEA tissue concentrations evaluated in tissue homogenates from stomach, duodenum, jejunum, ileum and colon in mice treated with pNAPE-LP + palmitate 0.0003 μg/kg or pLP + palmitate 0.0003 μg/kg by HPLC–MS. Results are expressed, for each two groups as the mean ± SD of *n* = 6 experiments performed in triplicate. A significantly increased tissue concentration of PEA was observed in the duodenum and ileum of pNAPE-LP + palmitate 0.0003 μg/kg-treated mice as compared to pLP + palmitate 0.0003 μg/kg (+200% and +148%, respectively, both * *p* < 0.05), while the highest tissue concentration was reached in the colon with a 123% increase vs. pLP + palmitate 0.0003 μg/kg (*** *p* < 0.0001).

In an in vitro preliminary analysis, we tested the actual presence of PEA in the supernatant of pNAPE-LP strains after the boost of an ultra-low dose of exogenous palmitate. PEA release was measured at 1, 3, 6, and 12 h after the exposure to exogenous palmitate; native *Lactobacillus paracasei* (pLP) served as the control. We observed a significant PEA release only when the culture medium was enriched with 0.0003 μg/mL of palmitate. The release of PEA reached the peak between 6 and 12 h, with a plateau detected at 12 h. In pLP, no detectable levels of released PEA were observed at the same time points, even when the medium was enriched with 0.0003 μg/mL of palmitate (Figure 1A). Paralleling the in vitro results, the intragastric administration of pNAPE-LP and palmitate for four consecutive days resulted in a significantly increased expression of PEA in the duodenum (0.27 ± 0.19, *p* < 0.05 vs. pLP + palmitate) ileum (0.44 ± 0.24, *p* < 0.05 vs. pLP + palmitate) and colon (1.62 ± 0.42, *p* < 0.001 vs. pLP + palmitate), as compared to pLP-treated mice, with the highest PEA concentrations achieved in distal colonic samples (+123% vs. pLP+ palmitate). On the contrary, no significant differences were observed in jejunal concentrations of PEA (Figure 1B).

### 2.2. Co-Administration of pNAPE-LP and Palmitate Improves the Severity of DSS-Induced Colitis in Mice 

**Figure 2:**
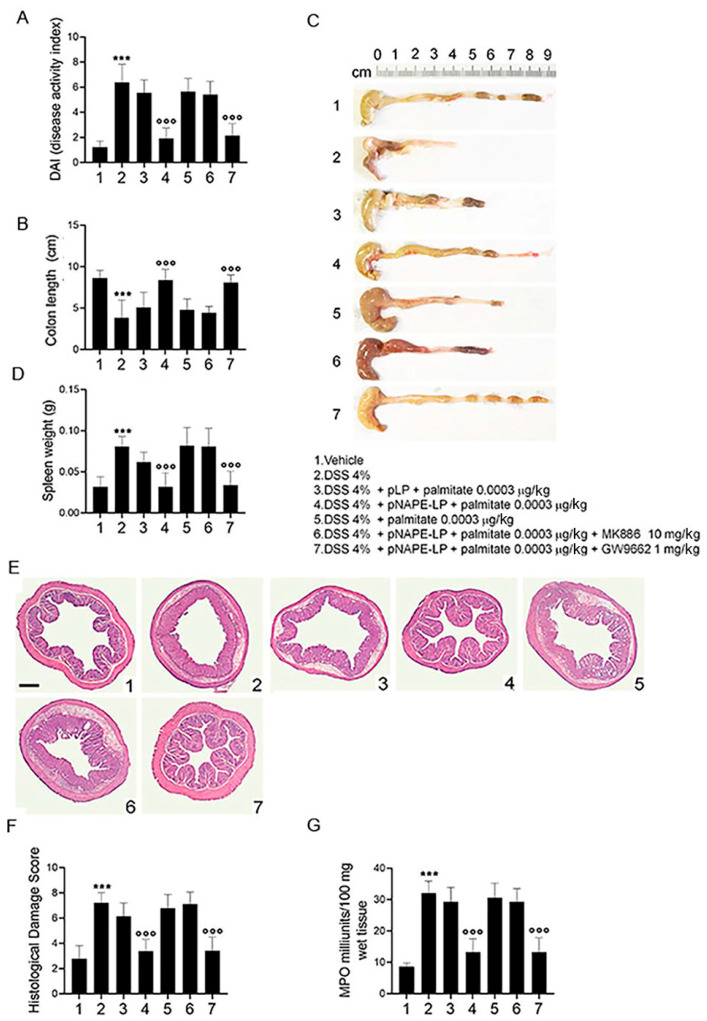
Engineered pNAPE-LP + palmitate ameliorates macroscopic signs of colitis, prevents colonic histological damage and neutrophil infiltration in DSS-treated mice. PPARα-dependent effects of pNAPE-LP + palmitate treatment on (**A**) DAI score, (**B**,**C**) colonic length and (**D**) spleen weight in DSS-exposed mice. (**E**) Representative images of hematoxylin and eosin (H&E) stained distal colon sections and (**F**) relative histological damage score showing the effect of pNAPE-LP + palmitate on DSS-induced colonic injury; magnification 4×; scale bar: 200 µm. (**G**) Myeloperoxidase (MPO) activity quantification as indirect evidence of neutrophils infiltration. Results are expressed as the mean ± SD of *n* = 5 experiments. *** *p* < 0.001 versus vehicle; °°° *p* < 0.001 versus DSS-treated mice.

Starting from day 4 after DSS administration (Figure 2), the disease activity index (DAI) score was significantly increased in colitis group (6.2 ± 1.45, *p* ˂ 0.001 vs. vehicle), with a marked raise in bloody diarrhea and a significant body weight loss, as compared to control mice (Figure 2A). Parallel to this, a significant colonic shortening, and an increased spleen weight were also observed (Figure 2B–D, 3.9 ± 2.13, 0.085 ± 0.012; all *p* ˂ 0.001 vs. vehicle). 

Co-administration of pNAPE-LP and palmitate (0.0003 μg/kg) significantly decreased DAI score, causing an overall improvement in the severity of all the above signs. A significant reduction in bloody diarrhea, an increase in body weight, an increase in colon length and a reduction in the spleen weight were indeed observed in mice receiving pNAPE-LP as compared to DSS-treated mice (Figure 2A–D, 1.8 ± 0.83, 8.3 ± 1.33, 0.032 ± 0.017; all *p* ˂ 0.001 vs. DSS). In mice receiving native *Lactobacillus Paracasei* (pLP), no significant changes in the severity of colitis were conversely observed, even in the presence of palmitate (Figure 2A–D). Additionally, administration of palmitate alone did not show any significant effect on DAI severity, colon length or spleen weight, confirming that palmitate per se did not affect the course of colitis. According to previously reported data [7,9,18], we also confirmed that the protective effects of pNAPE-LP + palmitate were almost completely abolished in the presence of the selective PPARα antagonist (MK886), but not the PPARγ antagonist (GW9662) (Figure 2A–D, 2.2 ± 0.83, 8.05 ± 0.95, 0.041 ± 0.017; *p* ˂ 0.001 vs. DSS), reflecting that pNAPE-LP-derived PEA exerts its beneficial effects through the selective involvement of PPARα receptors. 

### 2.3. pNAPE-LP and Palmitate Co-Administration Improves Colon Histopathological Damage, Mucosal Neutrophils Infiltration and Decreases Inflammatory Markers Expression and Release in DSS-Treated Mice

Histopathological analysis revealed severe mucosal damage in DSS-treated mice that was characterized by marked mucosal neutrophil infiltration and a significant increase in MPO activity (Figure 2E–G, 7.2 ± 0.79, 30.8 ± 4.6; *p* ˂ 0.001 vs. vehicle). The treatment with pNAPE-LP significantly ameliorated the colitis histopathological score and decreased MPO activity in comparison to DSS-treated mice (Figure 2E–G, 3.83 ± 0.95, 13.4 ± 4.16; *p* ˂ 0.001 vs. DSS). No significant effects on both mucosal inflammation and MPO activity were conversely observed in DSS-treated mice receiving pLP and palmitate co-administration, nor were pNAPE-LP alone or palmitate able to significantly improve mucosal damage and neutrophil infiltration (Figure 2E–G).

The protective effects of the pNAPE-LP strain were found to be dependent by selective targeting of PPARα receptors, because they were inhibited by selective PPARα, but not PPARγ antagonism (Figure 2E–G, 3.86 ± 1.11, 13 ± 4.6; *p* ˂ 0.001 vs. DSS). 

**Figure 3:**
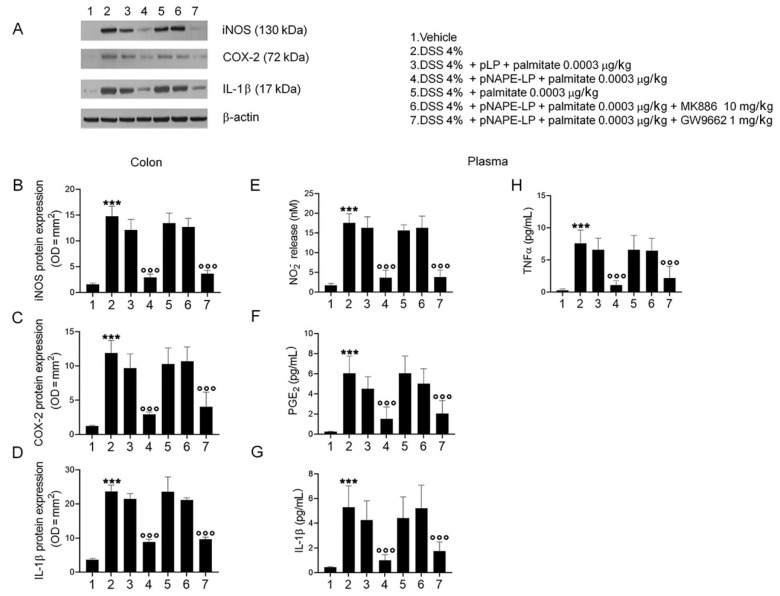
PEA release by pNAPE-LP + palmitate decreases pro-inflammatory mediators’ expression in the mouse colon and their release in the plasma through a selective PPARα involvement in DSS-treated mice. The administration of pNAPE-LP associated to palmitate (0.0003 μg/kg) induced a significant reduction in iNOS, COX-2 and IL-1β protein expression, as well as NO, PGE_2_, IL-1β and TNFα levels through PPARα-dependent involvement in mice colon and plasma. (**A**) Western blot analysis of iNOS, COX-2 and IL-1β protein expression and (**B**–**D**) relative densitometric analysis (arbitrary units normalized on the expression of the housekeeping protein β-actin). (**E**–**H**) Respective quantification of NO_2_^−^, PGE_2_, IL-1β and TNFα levels in mice plasma showing the effects of pNAPE-LP associated to palmitate (0.0003 μg/kg), given alone or in the presence of MK886 (10 mg/kg) or GW9662 (1 mg/kg) in the colonic tissue of DSS-treated mice. Results are expressed as the mean ± SD of *n* = 5 experiments performed in triplicate. *** *p* < 0.001 versus vehicle; °°° *p* < 0.001 versus DSS-treated mice.

The expression of pro-inflammatory signaling molecules and cytokines and their release were evaluated in colonic tissue homogenates and plasma samples, respectively. Our results demonstrated that DSS-treatment caused a marked increase in colonic iNOS, COX-2 and IL-1β in comparison to the vehicle group (Figure 3A–D, 14.9 ± 1.94, 11.6 ± 1.83, 24.1 ± 1.83; all *p* ˂ 0.001 vs. vehicle). Similarly, significant increases in the plasma level of NO, PGE_2_, IL-1β and TNF-α were observed (Figure 3E–H, 17.2 ± 2.35, 6.05 ± 1.7, 5.3 ± 1.73, 7.83 ± 2.08, respectively; all *p* ˂ 0.001 vs. vehicle). Treatment with pNAPE-LP and palmitate resulted in a significantly reduced expression and release of all the pro-inflammatory markers reported above, at both colonic and plasmatic levels (Figure 3A–D, 2.91 ± 0.64, 2.94 ± 0.31, 8.82 ± 0.81; Figure 3E–H, 3.64 ± 1.9, 1.55 ± 1.33, 1.01 ± 0.45, 1.05 ± 0.69; all *p* ˂ 0.001 vs. DSS). 

Again, the anti-inflammatory effects were significantly inhibited in the presence of the PPARα antagonist but not in the presence of the PPARγ antagonist (Figure 3A–D, 3.65 ± 0.64, 4.04 ± 2.13, 9.65 ± 0.6; Figure 3E–H, 4.83 ± 1.73, 2.05 ± 1.3, 1.74 ± 0.74, 2.1 ± 1.82; all *p* ˂ 0.001 vs. DSS), whereas administration of pNAPE-LP alone, palmitate, or pLP + palmitate failed to significantly inhibit the expression and the release of inflammatory mediators (Figure 3). 

### 2.4. pNAPE-LP and Palmitate Co-Administration Restores DSS-Induced Mucosal Integrity

**Figure 4:**
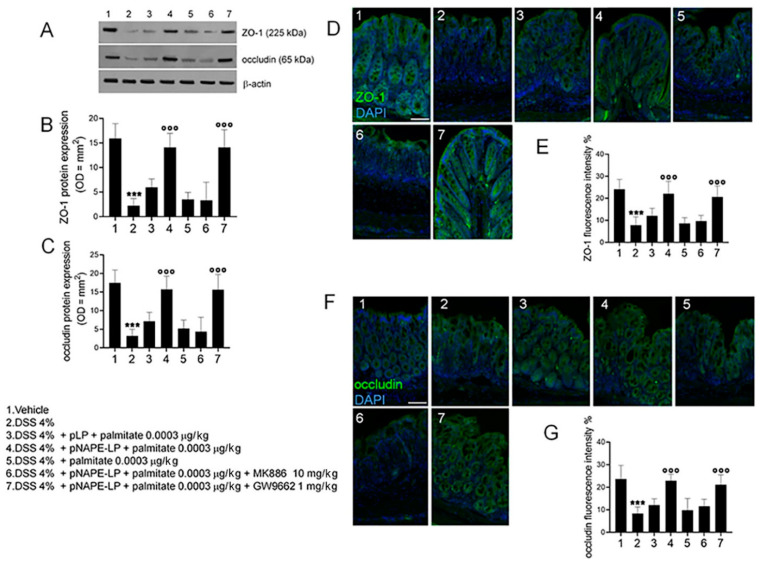
PEA released from pNAPE-LP + palmitate prevents the loss of tight junction proteins ZO-1 and occludin and colonic barrier disruption. (**A**) Immunoreactive bands and (**B**,**C**) relative densitometric analyses (arbitrary units normalized on the expression of the housekeeping protein β-actin), as well as immunofluorescence staining and their respective quantification corresponding to (**D**,**E**) ZO-1 and (**F**,**G**) occludin, showing the effects of pNAPE-LP combined to palmitate (0.0003 μg/kg), given alone or in the presence of MK886 (10 mg/kg) or GW9662 (1 mg/kg) on colonic mucosa of DSS-treated mice. Palmitate alone (0.0003 μg/kg) failed to significantly affect ZO-1 and occludin expression in colonic mucosa. Nuclei were also investigated using DAPI staining. Results are expressed as the mean ± SD of *n* = 5 experiments performed in triplicate. *** *p* < 0.001 versus vehicle; °°° *p* < 0.001 versus DSS-treated mice. Scale bar = 100 μm; magnification 10×.

Western blot and immunofluorescence analyses revealed a significant impairment of colonic mucosa integrity, as demonstrated by the significantly lower expression of zonula occludens (ZO-1) and occludin in DSS-treated, than in control mice (2.24 ± 1.44, 3.24 ± 1.75, 7.86 ± 3.69, 8.33 ± 2.87, respectively; both *p* ˂ 0.001 vs. vehicle; Figure 4). A marked recovery of mucosal integrity was observed in DSS-treated mice receiving pNAPE-LP + palmitate, with ZO-1 and occludin expression being significantly increased (14.4 ± 2.87, 16.1 ± 3.52, 22.3 ± 5.68, 24.9 ± 2.87; both *p* ˂ 0.001 vs. DSS; Figure 4). This effect was completely abolished by MK886, but not GW9662 (14.4 ± 3.58, 16.2 ± 4.09, 21.5 ± 4.85, 23.7 ± 4.33; both *p* ˂ 0.001 vs. DSS), further demonstrating the involvement of PPARα receptors, while the administration of pLP + palmitate or palmitate was not able to significantly improve mucosal integrity in DSS-induced mucosal damage (both *p* > 0.05 vs. DSS; Figure 4). 

## 3. Discussion

Our understanding of the pathophysiological role of gut microbiota underwent a paradigm shift in recent years. Considered as an innocent bystander for decades, accumulating evidence has clearly demonstrated its pivotal role in regulating several aspects of intestinal homeostasis, including mucosal integrity and inflammation [19,20]. In IBD, impaired host–microbiota interactions, resulting in a pro-inflammatory milieu, are essential for the maintenance and progression of mucosal inflammation [21,22]. The use of probiotics, by means of potential therapeutics in IBD [23], has therefore immediately captivated the scientific community as an innovative approach to control and inhibit gut inflammation [24]. However, despite the encouraging preclinical data, most probiotics are poor colonizers of the intestinal surface in vivo, and their bioactive metabolites are still poorly characterized. 

Aside from the implicit regulation of the host–microbiota imbalance postulated in IBD, probiotics offer the unique prospect of serving as potential delivery systems of anti-inflammatory molecules at the mucosal surface [25]. Genetically engineered probiotics able to colonize and express anti-inflammatory mediators in situ could overcome some of the current therapeutic failings, providing a novel efficient therapeutic approach in IBD [26]. 

In the pioneering work by Steidler et al., genetically modified *Lactococcus* (*Lc.*) *lactis*, expressing murine IL-10, was able to prevent colitis development in IL-10 KO mice and to improve inflammation in DSS-induced colitis [25]. This approach in humans was, however, hindered by the poor survival of this probiotic in the gastrointestinal tract, given its poor bile and acid resistance, and the authors suggested novel strategies, in order to improve the intestinal delivery of therapeutically engineered *Lc. lactis*, such as enteric coated formulations [26]. In a subsequent phase I clinical trial in Crohn’s patients, the enteric-coated engineered *Lc. lactis* has been shown to improve the disease course in humans [27]. However, in this clinical study, patients received both bile acid binders and proton pump inhibitors due to *Lc. lactis* poor viability, in order to improve the colonization of the GI tract. 

On the basis of such experimental paradigm, here, we demonstrated the feasibility of integrating a genetically-engineered probiotic, able to biosynthesize human NAPE-PLD, into the murine microbiota, and evaluated its effects on colonic inflammation in a well-validated mouse model of acute colitis, using *Lactobacillus paracasei* F19 spp., a widely used probiotic in clinical settings, that is featured by its peculiar genetic stability. 

*Lactobacillus* F19 has also been chosen for its favorable technological features: it can tolerate the gastric acidic environment (pH 2.5, 1 h) and exposure to bile (20%, 2 h), and hence has good ability to colonize and persist in the human intestine. Binding of collagen by *Lactobacillus* strains has been described earlier [28], which, combined with the absence of adverse effects during human trials, even in subjects with underlying disorders, suggests that pLP is safe and effective as a probiotic in humans [29,30]. 

In line with this, our data confirm that the colonization by pNAPE-LP is achieved after four days of treatment, and it results in the highest concentration of PEA in the distal colon. 

Our findings indicate that the oral treatment with pNAPE-LP and palmitate efficiently improves DSS-induced colitis in mice, as shown by the decreased DAI score, preservation of colonic length and the attenuation of splenomegaly. The co-administration of pNAPE-LP and palmitate also resulted in a significant histopathological improvement of colonic inflammation and neutrophil activation, as demonstrated by the reduced MPO activity. This, in turn, was mirrored by the significantly reduced expression and release of several proinflammatory molecules and cytokines. 

These potent anti-inflammatory effects were dependent on the pNAPE-LP ability of expressing the NAPE-PLD gene and producing PEA under the boost of ultra-low doses of exogenous palmitate. In fact, the administration of either pLP or palmitate alone was ineffective in counteracting colonic inflammation and improving colitis course. In parallel, PEA release caused an overall stabilization of mucosal barrier integrity in colitic mice, likely exploiting its well-known gate-keeper functions [7] due to PEA-induced up-regulation of ZO-1 and occludin proteins.

We also replicated previous data showing that these effects are secondary to PPARα receptors’ activation; the co-administration with PPARα, but not PPARγ antagonists, was able to almost completely prevent its anti-inflammatory effects, further providing indirect evidence of the key role PEA of in mediating pNAPE-LP effects. 

A number of genetically unmodified bacteria have shown potential anti-inflammatory properties in mice and, more recently, it has been proven that these effects are at least partially mediated by the endocannabinoid system. In a paper by Rossi et al., indeed, the widely used probiotic VSL#3 was able to modulate several genes encoding for enzymes involved in endocannabinoid (EC) metabolism and to relatively modulate the expression of CB1 and CB2 at the intestinal surface [31]. A clear advantage of using engineered pNAPE-LP rather than wild-type probiotics is the possibility of selecting carrier bacteria that can increase the likelihood of reaching therapeutic doses of the appropriate compound and selectively modulating the endocannabinoid system. 

Given its inability of activating the cannabinoid receptors, PEA is a very intriguing candidate-drug in IBD, because it offers the prospect of modulating the ECS without any virtual side effects [32,33]. A previous paper has also demonstrated that PEA is able to dose-dependently improve colonic inflammation both in mice and, most importantly, in human colonic tissue samples derived from patients with UC [7]. Thus, the main limiting factor to orally-administered PEA as a therapeutic in humans is largely related to its often-unpredictable tissue concentrations. The possibility of efficiently delivering and increasing the production of PEA in situ therefore represents a very promising strategy. Furthermore, PEA is a short-lived compound that is produced on demand and is rapidly metabolized to its inactive metabolites [34,35,36]. Several other strategies able to enhance PEA tissue delivery are under consideration, comprising the co-administration with polydatin and ultra-micronized formulation of PEA. However, given the short-lived activity of PEA, it is unclear whether any of these strategies could efficiently maintain its tissue concentration at therapeutic levels. 

A possible critical advantage of genetically engineered probiotic systems is that being able to adhere to the mucosal surface and colonize the gut for prolonged periods, they could serve as a sustained source of PEA produced in situ. Because PEA is a naturally occurring acylethanolamine, deriving from endogenous mammalians phospholipids, it seems highly unlikely to trigger an immune response, even when chronically biosynthesized by heterologous sources (i.e., gut microbiota) [37]. Furthermore, the fact that PEA production from the therapeutically engineered *Lactobacillus* is responsive to the co-administration of an exogenous substrate (palmitate), and that both PEA and pLP have a very favorable safety profile, with virtually no side effects observed in human trials, adds to the safety of our system. 

A limitation of our study is related to the fact that we did not explore the qualitative/quantitative changes in gut microbiota composition in mice. As previously stated, probiotics alone have shown the potential to modulate the ECS and positively impact on mucosal inflammation in IBD. However, pLP alone did not show any significant effects on mucosal inflammation in mice, and the pNAPE-LP + palmitate anti-inflammatory effects were mediated by the selective agonism at PPARα receptor sites, exerted by PEA release in vivo. One could argue that given the high plasticity of the acylethanolamine–PPARα axis, its anti-inflammatory effects could be attenuated for prolonged administrations. Further studies are required to determine the ideal interval and duration of booster administrations of pNAPE-LP able to maintain a sustained anti-inflammatory effect. 

Taken together, the results of the present study highlight the importance of pNAPE-LP as a new therapeutic tool that, by counteracting mucosal immune cells infiltration and proinflammatory mediators release, may improve colitis. Moving forward, further research to evaluate the long-term, ecological, and environmental safety of this genetically modified organism, is ongoing in order to possibly translate this approach in humans.

## 4. Materials and Methods

### 4.1. Generation of Genetically Modified Strains of Lactobacillus paracasei subsp. paracasei F19 

The pTRKH3-slpGFP vector (Addgene, Watertown, MA, USA) was first modified to remove the GFP sequence at SalI/PstI restriction sites, insert T7 transcriptional terminators at BamHI/EcoRV sites, and insert linker sequences containing BsaI-BsaI at PstI/XmaI restriction sites. The cDNA of human NAPE-PLD was then inserted into the BsaI sites using the In-Fusion method (Clontech, Mountain View, CA, USA). The resulting pTRKH3-slp-NAPE-PLD and parental plasmid (not expressing NAPE-PLD gene, used as negative control) constructs were transfected into the *Lactobacillus paracasei* subsp. *paracasei* F19 strain (Arla Foods, Hoersholm, Denmark) by electroporation, and positive clones were obtained by erythromycin (5 μg/mL) selection. Both parental plasmid (pLP) and NAPE-PLD-expressing bacteria (pNAPE-LP) were amplified anaerobically in Man, Rogosa and Sharpe (MRS)-broth (Conda, Torrejón de Ardoz Madrid, Spain) and isolated in MRS agar (Conda, Torrejón de Ardoz Madrid, Spain), both supplemented with erythromycin 5 μg/mL (Sigma-Aldrich, Milan, Italy) under anaerobic conditions for 72 h at 37 °C. Bacteria viability was determined by manually counting colonies, and the colony forming units (CFU)/mL were obtained through a colonies number correction for the dilution factor.

### 4.2. Animals and Experimental Design

Six-week-old male C57BL/6J mice (Charles River, Lecco, Italy) were used for the experiments. This gender/strain of rodents has been widely validated and investigated in DSS-induced colitis, given high animal susceptibility and detailed course in acute colitis [38]. All experimental procedures were approved by Sapienza University’s Ethics Committee. Animal care was in compliance with the IASP and European Community (EC L358/1 18/12/86) guidelines on the use and protection of animals in experimental research. Mice were randomly divided into the following groups (*n* = 10 each): (1) non-colitic (vehicle) group; (2) colitic group receiving a daily intragastric gavage with 200 µL MRS broth without probiotic supplementation; colitic groups receiving a daily intragastric gavage with either (3) pLP or (4) pNAPE-LP combined with palmitate (0.0003 μg/kg); and (5) colitic group receiving a daily intragastric gavage with palmitate alone (0.0003 μg/kg), colitic groups receiving a daily intragastric gavage with pNAPE-LP combined with palmitate (0.0003 μg/kg) in the presence of selective (6) PPARα antagonist MK886 (10 mg/Kg) or (7) PPARγ antagonist GW966 (1 mg/Kg), respectively. A representative figure of our experimental plan is depicted in Figure S1A. 

In all groups, colitis was experimentally induced by administering dextran sulfate sodium (DSS 4% *w*/*v*, MW 36,000 to 50,000, Sigma Aldrich, Italy) in drinking water for six consecutive days (starting from day 1). Probiotic treatment was given daily from day 2 until day 6 by intragastric administration of 0.1 mL of bacteria suspension containing 0.8–1.2 × 10^9^ CFU/mL of pLP or pNAPE-LP together with palmitate 0.0003 μg/kg. PPARα antagonist MK886 and PPARγ antagonist GW966 were given intraperitoneally from day 2 to day 6. During the whole length of the study, animal body weight, stool consistency and presence of bloody diarrhea were recorded daily to determine the disease activity index (DAI) (see Figure S1B). Animals were sacrificed at day 7 after colitis induction, spleen weight and colon length were measured after post-mortem isolation, and colonic tissues were removed to perform macroscopic, histochemical, and biochemical analyses, as described below. 

### 4.3. In Vitro and In Vivo Quantification of Bacteria-Produced PEA by HPLC–MS Method

Specimens from the stomach, duodenum, jejunum, ileum, and distal colon from a subset of mice of the vehicle group treated with 0.1 mL of bacteria suspension containing 0.8–1.2 × 10^9^ CFU/mL of pLP or pNAPE-LP together with palmitate 0.0003 μg/kg were isolated to evaluate PEA concentrations in vivo (*n* = 12 in total, 6 mice treated with pLP and 6 mice treated with pNAPE-LP). Tissues were processed according to the method described by the Endocannabinoid Research Group [39]. Extraction and analysis were performed according to Gachet et al. [40], with slight modifications. Firstly, samples of bacterial cultures were ultra-centrifuged at 10,956× *g* for 10 min, obtaining a supernatant (representing the culture medium) and a pellet (representing the bacteria). An amount of 250 µL of supernatant was extracted with the same volume of acetonitrile (ACN) with 0.1% formic acid (extraction solution), vortexed for 1 min, and placed at 4 °C for 10 min, to facilitate the precipitation of proteins. Then, the samples were centrifuged (10,956× *g*, 4 °C, 5 min) and the supernatant was injected for the mass spectrometry analysis. For the lysis of the bacterial pellet, 200 µL of extraction solution were added to each sample and vortexed for 1 min. Samples were kept to −20 °C for 10 min and then in an ultrasound bath for a total of 30 min (2 cycles of 15 min each, with 5 min of break). Subsequently, the samples were centrifuged (10,956× *g*, 4 °C, 5 min) and the supernatant was injected for the mass spectrometry analysis. Analyses were run on a Jasco Extrema LC-4000 system (Jasco Inc., Easton, MD, USA) coupled to an Advion Expression mass spectrometer (Advion Inc., Ithaca, NY, USA) equipped with an electrospray (ESI) source. Mass spectra were recorded in positive SIM mode. The capillary voltage was set at +180 V, the spray voltage was at 3 kV, the source voltage offset was at +20 V, and the capillary temperature was set at 250 °C. The chromatographic separation was performed on a Kinetex C18 analytical column (150 × 4.6 mm, id. 3 µm, 100 Å) and security guard column, both supplied by Phenomenex (Torrance, CA, USA). The analyses were performed at a flow rate of 0.3 mL/min, with solvent A (water containing 2 mM ammonium acetate) and solvent B (methanol containing 2 mM ammonium acetate and 0.1 % formic acid). Elution was performed according to the following linear gradient: 15% B for 0.5 min, 15–70% B from 0.5 to 2.5 min, 7–99% B from 2.5 to 4.0 min and held at 99% B from 4.0 to 8.0 min. From 8 to 11.50 min, the column was equilibrated to 15% B and conditioned from 11.5 to 15.0 min at 15% B. The injection volume was 10 µL and the column temperature was fixed at 40 °C. For quantitative analysis, standard curves of PEA (Sigma-Aldrich St. Louis, MO, USA) were prepared over a concentration range of 0.0001–1 ppm with six different concentration levels and duplicate injections at each level. All data were collected and processed using JASCO ChromNAV (v2.02.04) and Advion Data Express (v4.0.13.8).

### 4.4. Disease Activity Index (DAI)

The DAI scale was used to evaluate experimental colitis induction and progression. DAI was determined by scoring changes in body weight (0 = none; 1 = 1 to 5%; 2 = 5 to 10%; 3 =10 to 20%; 4 = >20%); stool consistency (0 = normal; 2 = loose; 4 = diarrhea) and rectal bleeding (0 = normal; 2 = occult bleeding; 4 = gross bleeding), according to the criteria proposed by Cooper et al. [41]. DAI score was recorded daily (from day 0 to day 7) and the results were expressed as cumulative average scores in each experimental group.

### 4.5. Histopathological Analysis

After sacrifice, mouse distal colons were fixed in 4% paraformaldehyde (PFA), sectioned into 15 μm slices, and stained with hematoxylin and eosin (H&E) for macroscopic and histopathological assessment. Colonic histological damage was evaluated through a complex score, according to the criteria proposed by Li et al. [42] considering the following parameters: (i) distortion and loss of crypt architecture (0 = none; 1 = mild; 2 = moderate; 3 = severe); (ii) infiltration of inflammatory cells (0 = normal; 1 = mild infiltration; 2 = moderate infiltration; 3 = dense infiltration); (iii) muscle thickening (0 = normal; 1 = mild muscle thickening; 2 = moderate muscle thickening; 3 = marked muscle thickening); (iv) goblet cell depletion (0 = absence; 1 = presence); (v) crypt absence (0 = absence; 1 = presence). Slices were analyzed with a microscope Nikon Eclipse 80i by Nikon Instruments Europe (Nikon Corporation, Tokyo, Japan), and images were captured at 4× magnification by a high-resolution digital camera (Nikon Digital Sight DS-U1). Cumulative histological damage scores were expressed as average scores in each experimental group. 

### 4.6. Protein Extraction and Western Blot Analysis

Proteins were extracted from colonic tissue and processed by Western blot analysis. Briefly, the samples were homogenized in ice-cold hypotonic lysis buffer (10 mM 4-(2-hydroxyethyl)-1-piperazineethanesulfonic acid (HEPES), 1.5 mM MgCl_2_, 10 mM KCl, 0.5 mM phenylmethylsulphonylfluoride, 1.5 mg/mL soybean trypsin inhibitor, 7 mg/mL pepstatinA, 5 mg/mL leupeptin, 0.1 mM benzamidine and 0.5 mM dithiothreitol (DTT)). The resulting cytosolic extracts were mixed with a non-reducing gel loading buffer (50 mM Tris (hydroxymethyl) aminomethane (Tris), 10% sodiumdodecylsulfate (SDS), 10% glycerol, 2 mg bromophenol/mL) at a 1:1 ratio, and then boiled for 3 min followed by centrifugation at 10,000× *g* for 10 min. Protein concentration was determined using Bradford assay and equivalent amounts (50 μg) of each homogenate underwent electrophoresis through a polyacrylamide minigel. Proteins were transferred to nitrocellulose membranes that were saturated by incubation with 10% non-fat dry milk in 1X PBS overnight at 4 °C and then incubated with either rabbit polyclonal anti-iNOS (Novus Biological, Abingdon, UK), rabbit polyclonal anti-COX-2 (Cell Signaling Technology, Danvers, MA, USA), rabbit polyclonal anti-IL-1β, rabbit polyclonal anti ZO-1, rabbit monoclonal anti-occludin (Abcam, Cambridge, UK) or mouse monoclonal anti-β-actin (Santa Cruz Biotechnology, CA, USA) for 2 h at room temperature (RT). Membranes were then incubated with the specific secondary antibodies conjugated to horseradish peroxidase (HRP) (Dako, Milan, Italy). Immune complexes were revealed by enhanced chemiluminescence detection reagents (Amersham Biosciences, Milan, Italy) and exposed to Kodak X-Omat film (Eastman Kodak Co., Rochester, NY, USA). Protein bands were then scanned and densitometrically analyzed with a GS-700 imaging densitometer. Results were expressed as OD (arbitrary units; mm^2^) and normalized on the expression of the housekeeping protein β-actin for mice and bacterial proteins, respectively.

### 4.7. Preparation of Blood Samples

Before being sacrificed, mice were deeply anesthetized. Blood samples were taken by cardiac puncture and collected in 5% EDTA vials, immediately prior to sacrifice. To determine nitric oxide (NO), prostaglandin E2 (PGE2), interleukin 1β (IL-1β) and tumor necrosis factor-α (TNFα) levels, plasma was then isolated from the blood, immediately frozen, and stored at −80 °C until the assays.

### 4.8. Enzyme-Linked Immunosorbent Assay for TNFα, PGE_2_ and IL-1β

Enzyme-linked immunosorbent assay (ELISA) for PGE_2_, IL-1β and TNFα (all Thermo Fisher Scientific, MA, USA) was carried out on mouse plasma according to the manufacturer’s protocol. Absorbance was measured on a microtiter plate reader. PGE_2_, IL-1β and TNFα levels were determined using standard curve methods.

### 4.9. NO Quantification

NO production was measured as nitrite (NO_2_^−^) accumulation in murine plasma by a spectrophotometer assay based on the Griess reaction [43]. Briefly, Griess reagent (1% sulphanilamide, 0.1% naphthylethylenediamine in H_3_PO_4_) was added to an equal volume of plasma and the absorbance was measured at 550 nm. Nitrite concentration (nM) was thus determined using a standard curve of NaNO_2_.

### 4.10. Myeloperoxidase Activity 

Myeloperoxidase (MPO) activity was evaluated in colonic tissues to determine the extent of neutrophil infiltration and activation, as previously described [44]. After removal, mice colonic tissues were rinsed with a cold saline solution, opened, and deprived of the mucosa using a glass slide. The resulting layer was then homogenized in a solution containing 0.5% hexadecyltrimethylammonium bromide (Sigma-Aldrich) dissolved in 10 mM potassium phosphate buffer and centrifuged for 30 min at 20,000× *g* at 37 °C. An aliquot of the supernatant was mixed with a solution of tetramethylbenzidine (1.6 mM; Sigma-Aldrich) and 0.1 mM hydrogen peroxide (Sigma-Aldrich). The absorbance was then spectrophotometrically measured at 650 nm. MPO activity was determined as the amount of enzyme degrading 1 mmol/min of peroxide at 37 °C and was expressed in milliunits per 100 mg of wet tissue weight.

### 4.11. Immunofluorescence Analysis 

On day 7, animals were sacrificed, and distal colon was isolated then fixed in ice-cold 4% paraformaldehyde (PFA) and sectioned into 20 μm slices. Sections were blocked with bovine serum albumin and subsequently stained with rabbit anti-ZO-1 antibody (1:100 dilution *v*/*v*; Proteintech, Manchester, UK) or rabbit anti-occludin antibody (1:100 dilution *v*/*v*; Novus Biologicals, Abingdon, UK). Slices were then washed with PBS 1X and incubated in the dark with fluorescein isothiocyanate-conjugated anti-rabbit (Abcam, Cambridge, UK). Nuclei were stained with Hoechst. Sections were analyzed with a microscope (Nikon Eclipse 80i), and images were captured by a high-resolution digital camera (Nikon Digital Sight DS-U1).

### 4.12. Statistical Analysis

Results were expressed as the mean ± SD of experiments. Statistical analysis was performed using parametric one-way analysis of variance (ANOVA) and multiple comparisons were performed by Bonferroni’s post hoc test. *p*-values < 0.05 were considered statistically significant.

## Data Availability

The raw data supporting the conclusions of this article will be made available by the authors, without undue reservation.

## Supplementary Materials

The supplementary materials are available online at https://www.mdpi.com/1422-0067/22/6/2945/s1.
