# Supplementary material for: Engineered Lactobacillus paracasei Producing Palmitoylethanolamide (PEA) Prevents Colitis in Mice
Source: Int J Mol Sci. 2021 Mar 14;22(6):2945. doi: 10.3390/ijms22062945 (PMC7999950; doi:10.3390/ijms22062945)
Supplement: Supplementary file 1 [file ijms-22-02945-s001.pdf]

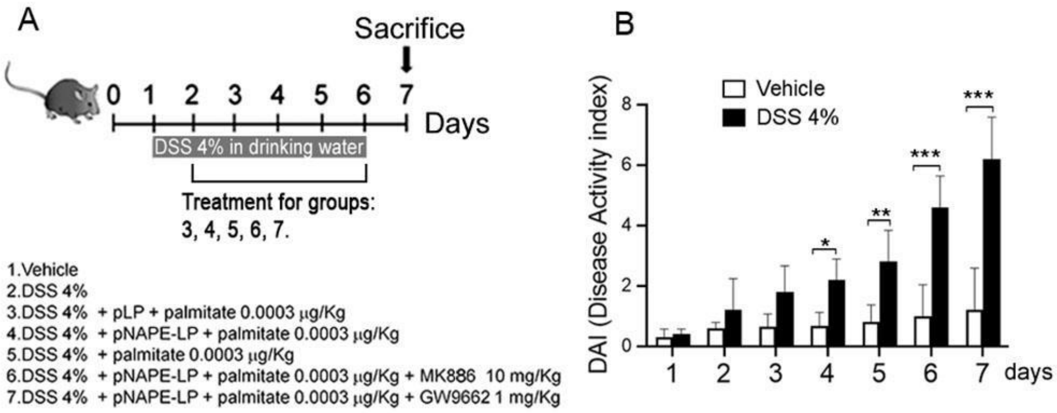

Figure S1. Experimental plan and colitis induction (**A**) Representative scheme showing our experimental protocol. Vehicle group received no treatment and served as internal control. All other mice groups were administered dextran sulfate sodium (DSS 4% w/v, MW 36,000 to 50,000, Sigma Aldrich, Italy) in drinking water for six consecutive days (starting from day 1). Starting from day 2, mice were randomly divided into the following groups (*n* = 10 each): (1) no further treatment (DSS 4%); (2) pLP+ palmitate (0.0003 μg/Kg), (3) pNAPE-LP + palmitate (0.0003 μg/Kg) (4) palmitate alone (0.0003 μg/Kg) (5) pNAPE-LP + palmitate (0.0003 μg/Kg) with the selective PPARα antagonist MK886 (10 mg/Kg) and (6) pNAPE-LP + palmitate (0.0003 μg/Kg) with the selective PPARγ antagonist GW966 (1 mg/Kg), respectively. All treatments were given daily from day 2 until day 6 by intragastric gavage, while PPARα and PPARγ antagonists were administered intraperitoneally from day 2 to day 6. (**B**) Kinetic of colitis induction showing DAI measurements from day 0 to 7 in DSS-treated mice.
